# Targeting myeloperoxidase to stabilize unruptured aneurysm: an imaging-guided approach

**DOI:** 10.1186/s12872-024-03822-1

**Published:** 2024-03-20

**Authors:** Xingchi Shi, Yuan Xue, Huiyu Wu, Chengyi Shen, Lei Zhong, Jun Lei, Zhiyang Xia, Ying Yang, Jiang Zhu

**Affiliations:** 1 Medical Imaging Key Laboratory of Sichuan province, Department of Oncology, Affiliated Hospital of North Sichuan Medical College, Maoyuan Road 1, Nanchong City, 637000 Sichuan China; 2 Department of Cardiovascular disease, School of Clinical Medicine, Affiliated Hospital of North Sichuan Medical College, Maoyuan Road 1, Nanchong City, 637000 Sichuan China; 3 Institute of Basic Medicine and Forensic Medicine, North Sichuan Medical College, Fujiang Road 234, Nanchong City, 637000 Sichuan China; 4 School of Pharmacy, North Sichuan Medical College, Fujiang Road 234, Nanchong City, 637000 Sichuan China

**Keywords:** Myeloperoxidase, Aneurysm, Mn-TyrEDTA, 4-aminobenzoic acid hydrazide, Metalloproteinases

## Abstract

Inflammation plays a key role in pathogenesis and rupture of aneurysms. Non-invasively and dynamically monitoring aneurysm inflammation is critical. This study evaluated myeloperoxidase (MPO) as an imaging biomarker and therapeutic target for aneurysm inflammation using an elastase-induced rabbit model treated with or without 4-aminobenzoic acid hydrazide (ABAH), an irreversible inhibitor of MPO. Myeloperoxidase-sensitive magnetic resonance imaging (MRI) using Mn-TyrEDTA, a peroxidase activity-dependent contrast agent, revealed weak contrast enhancement in contralateral arteries and decreased contrast enhancement in aneurysm walls with ABAH treatment, indicating MPO activity decreased and inflammation mitigated. This was supported by reduced immune cell infiltration, matrix metalloproteinases (MMP-2 and − 9) activity, ROS production and arterial wall destruction on histology. Finally, the aneurysm expansion rate remained < 50% throughout the study in the ABAH(+) group, but increased gradually in the ABAH(-) group. Our results suggest that inhibition of MPO attenuated inflammation and expansion of experimental aneurysm and MPO-sensitive MRI showed promise as a noninvasive tool for monitoring aneurysm inflammation.

**Supplementary Information:**

The online version contains supplementary material available at 10.1186/s12872-024-03822-1.

## Introduction

Intracranial Aneurysm (IA) can be a devastating event with an estimated prevalence of 2% in the general population and a 1-year mortality of 65–90% for untreated ruptured IA [1,2]. Based on Western population-based studies, ruptured IA accounts for 1–7% of all strokes [3], leading to long-term disability and enormous emotional and socioeconomic burdens [4]. A better understanding of IA pathogenesis could improve patient outcomes.

Recent evidence indicates that inflammation plays a key role in the major pathological changes of IAs, including recruitment and infiltration of inflammatory cells (neutrophils, macrophages, T and B lymphocytes, dendritic cells, mast cells [5,6]), vascular smooth muscle cells apoptosis, elastin and collagen degradation, and extracellular matrix (ECM) breakdown. Theses changes lead to arterial dilation, bulging, and even rupture [7–9]. Non-invasive and dynamic monitoring of inflammatory status in the aneurysm wall may provide critical information to guide IA management, especially in high- risk patients.

Myeloperoxidase (MPO) is a heme protein found in the azurophilic granules of neutrophils and monocytes. Harmful stimuli like pathogens, damaged cells, or irritants can trigger self-protective responses of inflammation and activate these phagocytes to release MPO, which produces high toxic various of reactive oxidative species (ROS), such as hypochlorous acid (HOCl) and free radicals [10,11]. However, excess MPO-derived oxidants during sustained oxidative stress have been linked to oxidative tissue damage in many chronic and acute inflammatory diseases [12–15].

In aneurysms lesion, matrix metalloproteinases (MMPs) and tissue inhibitors of matrix metalloproteinases (TIMPs) are essential for degrading the extracellular matrix (ECM). The potent oxidant HOCl, derived from MPO, can activate MMPs by converting the thiol residue in their inactive zymogen to a sulfinic acid and inactivate TIMPs by oxidizing their N-terminal cysteine residue [16–19]. Therefore, MPO-mediated imbalance of MMPs and TIMPs leads to degradation of various protein substrates in the ECM, including collagen and elastin [20,21].

Moreover, MPO-derived oxidative species strongly correlate with endothelial dysfunction, phenotypic switching of vascular smooth muscle cell (VSMCs), and ERK1/2 signaling activation [22–25], suggesting a potential link between aneurysm pathogenesis and MPO. Overall, MPO could be explored as an imaging biomarker and potential therapeutic target for IA.

**Fig. 1:**
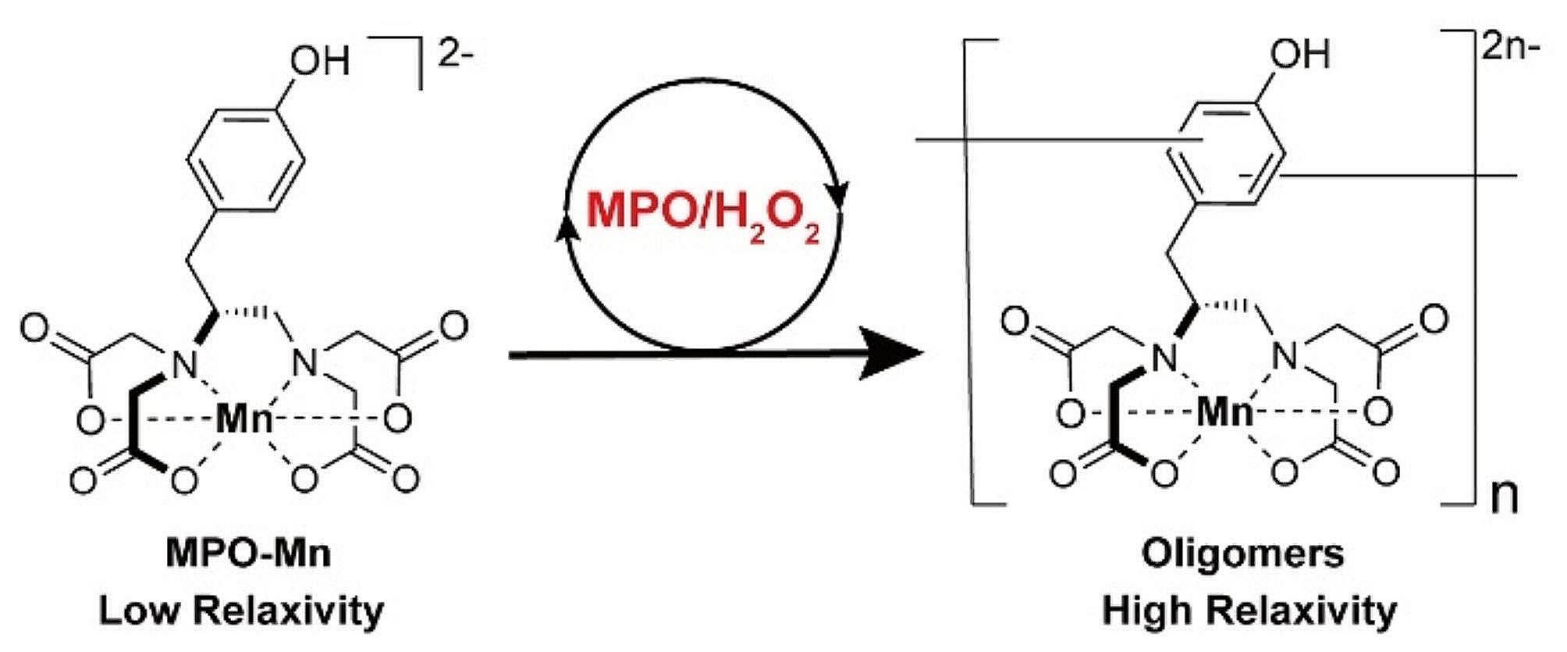
Peroxidase-activatable Mn(II)-based MRI molecular probe (Mn-TyrEDTA) with peroxidase activity-dependent relaxivity

As shown by an earlier investigation, an Mn(II)-based MRI probe, Mn-TyrEDTA, containing an L-tyrosine-derived ligand with a phenol group can serve as an electron donor and substrate for MPO/H_2_O_2_ to oligomerize and amplify MR signal (Fig. 1) [26]. With its peroxidase activity-dependent relaxivity, Mn-TyrEDTA can produce strong contrast enhancement selectively in inflamed tissues with high MPO expression in an MSU-induced mouse model of acute gout.

In this study, we created carotid aneurysms in 59 New Zealand White rabbits to explore the role of MPO in a preclinical model. Carotid aneurysms could mimic the morphologic features [27], histopathologic findings [28], and hemodynamic features [29] of human IAs. Using MPO as the imaging biomarker, we dynamically and non-invasively assessed the inflammatory state of aneurysms through MPO-sensitive MR imaging. To evaluated the effects of MPO inhibition on aneurysm growth, wall inflammation, and healing, rabbits were treated with 4-aminobenzoic acid hydrazide (ABAH, an irreversible inhibitor of MPO which has been used in vivo for many diseases, including subacute stroke [30], nonalcoholic steatohepatitis [31], ischemic stroke [32], multiple sclerosis [33]). Further, we explored the underlying mechanism through MPO activity assay, immunofluorescence assay, ROS detection and MMPs activity assay.

## Experimental

### Study design

To evaluate the inflammatory response in a rabbit model of aneurysm, carotid aneurysms were created in 59 New Zealand White rabbits. Animals were provided by Animal Experimental Center of North Sichuan Medical College, and randomly allocated to one of two groups as ABAH(+) (*n* = 31), or ABAH(-) (*n* = 28) using a computer-generated table of random numbers. Two groups were assigned to a study with or without 4-aminobenzoic acid hydrazide (ABAH, an irreversible inhibitor of MPO [30–33]) respectively. Primary outcomes were defined as aneurysm wall signal intensity and aneurysm growth of 4 consecutive weeks after its creation based on MR imaging findings. Secondary outcomes were aneurysm wall histology, MPO activity and expression, and MMPs activity at week 1 and 4 after intervention.

**Fig. 2:**
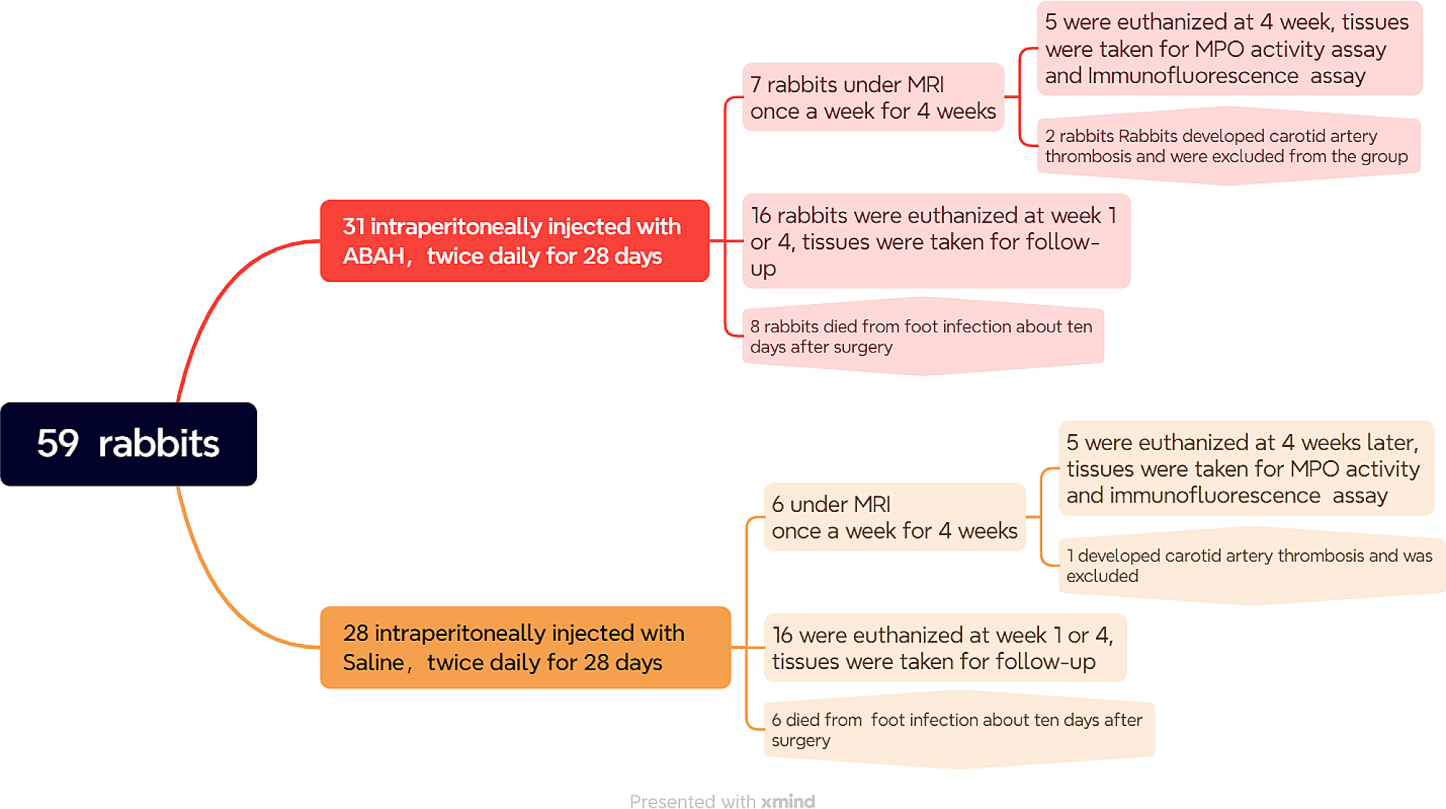
Flow chart of study population

The 59 male rabbits with a mean weight of 2.5 ± 0.5 kg and mean age of 16 weeks (± 3days) were housed at room temperature of 22–24 °C with a 12-hour light/dark cycle. The rabbits had free access to a hay diet as well as water. All animals received daily care in accordance with the local institutional guidelines. Animals allocated to the ABAH(+) groups were administered intraperitoneally with ABAH (40 mg/kg), twice daily [33] for 28 days, with same dose of saline to the ABAH(-) group. Rabbits with carotid artery embolism (which is detected in the subsequent MR imaging) or who is dead during the 4 follow-up weeks were excluded from the analysis. Figure 2, shown below, summarized the overall process.

### Carotid aneurysm model and surgical technique

Detailed technique of surgical creation of the carotid aneurysm have been described elsewhere [34]. Briefly, for rabbits undergoing surgery, anesthesia was induced with 4% isoflurane gas in a gas chamber, and maintained with 2% isoflurane via a face mask. Carotid aneurysms were created with 30 units of porcine pancreatic elastase (sigma, USA) retaining in the left common carotid artery for 20 min. All animals were given daily clopidogrel (1 mg/kg), heparin (100 U) for anti-coagulation [35], and penicillin (200,000 U) for anti-infection treatment for 7 days.

### Contrast medium

In this study, the inflammatory response was assessed by contrast-enhanced T1-weighted imaging using the MPO-activatable contrast agent Mn-TyrEDTA, which was prepared using a previously reported protocol [26]. The T1-relaxivity of Mn-TyrEDTA increases with increasing peroxidase activity (at 0.47 T, 32 °C, horseradish peroxidase (HRP) = 0 U, *r*_1_ = 3.3 mmol^[-1^s^[-1^; HRP = 500 U, *r*_1_ = 8.2 mmol^[-1^s^[-1^).

### MPO-sensitive MR imaging

All animals were examined on a 3.0 T MR scanner (Discovery MR750, GE Medical System, Milwaukee, WI) with a Knee Coil under respiration-monitored. For rabbits undergoing MRI, anesthesia was induced with 4% isoflurane gas, and maintained at 2% isoflurane. Three-dimensional time of flight (3D TOF) MRA and T2-weighted (T2W) scans were performed to locate and evaluate the overall condition of the aneurysm before injection of the contrast agent. MPO-sensitive molecular MR imaging was performed to evaluate MPO activity within the lesion using Mn-TyrEDTA as the probe at a dosage of 0.1 mmol/kg injected intravenously at the ear margin in both groups of rabbits (*n* = 5). The duration of the acquisitions is 20 min. T1-weighted imaging (T1WI) was performed before and 0 min, 5 min, 10 min, 15 min, and 20 min post-injection of the probe, respectively. The sequence parameters are as follows: 3D TOF TR / TE = 22 ms / 5.7 ms; matrix = 320 × 256; flip angle (FA) = 20°; section thickness = 0.6 mm; bandwidth = 31.20 kHz; field of view (FOV) = 20 × 20 cm [2]; number of excitations (NEX = 1). T2-weighted FSE Parameters(with fat-suppression)TR / TE = 4241 ms / 72 ms; matrix = 288 × 192; flip angle (FA) = 142°; section thickness = 1 mm; bandwidth = 31.76 kHz; field of view (FOV) = 18 × 18 cm [2]; number of excitations (NEX = 3). T1-weighted FSE Parameters (with fat-suppression) TR / TE = 775 ms / 15 ms; matrix = 320 × 224; flip angle (FA) = 142°; section thickness = 1 mm; bandwidth = 31.65 kHz; field of view (FOV) = 10 × 10 cm [2]; number of excitations (NEX = 3).

The acquired images are transferred to a workstation (Advantage Workstation 4.4; GE Healthcare) and analyzed by an investigator who was blinded to the experimental groups. Regions of interest (ROIs) were then drawn manually on the slice showed the most enlarged arterial lumen for quantitative analysis. Signal intensity (SI) of the left carotid artery, the right carotid artery and adjacent muscle was measured before and after injection of contrast agents, and the noise was defined as the standard deviation (SD) of the signal in the air. Using the adjacent muscle as control, contrast-to-noise ratio (CNR) of the left aneurysmal lesion or right normal artery was calculated using the following formula: CNR = (SI_left or right_ - SI muscle)/SD_air_; the enhancement attributable to MPO was then assessed by calculating the ΔCNR = CNR_post-contrast_ - CNR_pre-contrast_. Vascular expansion rate = vascular diameter (largest on the ipsilateral) - vascular diameter (contralateral) / vascular diameter (contralateral position) × 100%.

### Histology

Histology tests were performed by an investigator who was blinded to the experimental groups on two cohorts of ABAH (-) (*n* = 6; receiving saline vehicle) and ABAH (-) (*n* = 6) rabbits on days 7 and 28 after surgical creation of the carotid aneurysm. Rabbits were euthanized by an intraperitoneal overdose ( > = 100 mg/kg) of sodium pentobarbital. Carotid aneurysms were identified optically, isolated and dissected (with the contralateral artery as control). The tissue was fixed with 4% paraformaldehyde, embedded in paraffin (2–3 μm) and stained with Hematoxylin and eosin (HE) staining for overall histology. Neutrophils and macrophages were counted by professional pathologists. Masson staining and Elastin Van Gieson (EVG) staining were used to evaluate the content and integrity of collagen and elastic fibers of carotid aneurysms, respectively.

### MPO activity assay

The MPO activity assay is based on the previously described method [36]. The carotid aneurysm was weighed, ground and homogenized in 0.5% sodium cetyl sulfate buffer, and then the homogenate was frozen/thawed four times, followed by centrifugation at 13,000 g at 4 °C for 20 min to obtain its supernatant. 10ul of supernatant was added to 3.0 ml of a solution which prepared by the following solution containing: 26.9 ml of water, 3.0 ml of sodium phosphate buffer solution (0.1 M, pH 7.0), 0.1 ml of H_2_O_2_ (0.1 M), and 0.048 ml of guaiacol. The optical density change(ΔOD) per minute is calculated from the second minute absorbance rate. The peroxidase activity was calculated according to the following equation: Activity = (OD × Vt × 4) / (E × t × Vs), E = 26.6 mM^[-1^ cm^[-1^ at 470 nm; OD = change in absorbance; Vt = total volume; Vs = sample volume and t = change in time. After measuring the total protein content in each sample, the results were expressed as U of MPO activity per gram of tissue. Horseradish peroxidase was used as a positive control.

### Immunofluorescence detection of MPO

The rabbit carotid aneurysm tissue, prepared as described earlier, were de-paraffinized, rehydrated and subjected to antigen retrieval. The sections were then incubated with rabbit anti-MPO (1:100, Bioss antibody, bs-4943R) overnight at 4 °C, FITC-labeled goat anti-rabbit secondary antibody (1:100, Servicebio, GB22303)was added and incubated at room temperature for 30 min. DAPI (ZSGB-BIO, ZLI-9557) was added at room temperature for 10 min to delineate nucleus. The sections were imaged using a microscopic camera system (3DHISTECH, CaseViewer 2.4).

### ROS detection

One week postoperatively, the rabbits were euthanized. The carotid aneurysms were identified visually, isolated, dissected, and prepared as frozen sections as previous study demonstrated [37]. The contralateral artery incubated with ROS detecting kit build-in Rosup was used as a positive control. ROS production in aneurysms was detected by treating frozen section of arterial tissue with 10 µM of 2’,7’-dichlorodihydrofluorescein diacetate (DCFH-DA; Beyotime Biotechnology) and observing the fluorescence of DCFH (Ex: 488 nm, Em: 525 nm).

### Gelatin zymogram

The activities of MMP-2 and MMP-9 in rabbit carotid aneurysms were detected by a MMP Zymography Assay Kit (Applygen). The protein sample obtained from arteries were loaded on to a gelatin-coated pre-casted polyacrylamide gel (Bio Rad). Electrophoresis was carried out under sodium dodecyl sulfate (SDS) at constant voltage. Gel was incubated with 2.5% Triton X-100 at room temperature for 2 h to remove SDS. Then the gel was washed 3 times to remove Triton X-100 and incubated at 37 ℃ in a developing buffer (Tris-HCl, pH 7.4) containing 10mM CaCl_2_. 24 h later, the gel was stained with Coomassie Brilliant Blue R250. The metalloprotease activity was visualized after de-staining and then photographed. The intensity of the bands was quantitated by densitometry. Rabbit whole blood was used as a positive control.

### Statistical analysis

All statistical analyses were calculated using the unpaired student’s t-test after F-test and S-W test were conducted and met. A p value of < 0.05 was considered significant. The data were statistically analyzed by SPSS 26 statistical analysis software. ImageJ (version 1.45s, National Institutes of Health, MD, USA) was used for image analysis. All values are expressed as mean ± SD.

## Results

### Mn-TyrEDTA reveals the reduced contrast enhancement of aneurysm lesion

**Fig. 3:**
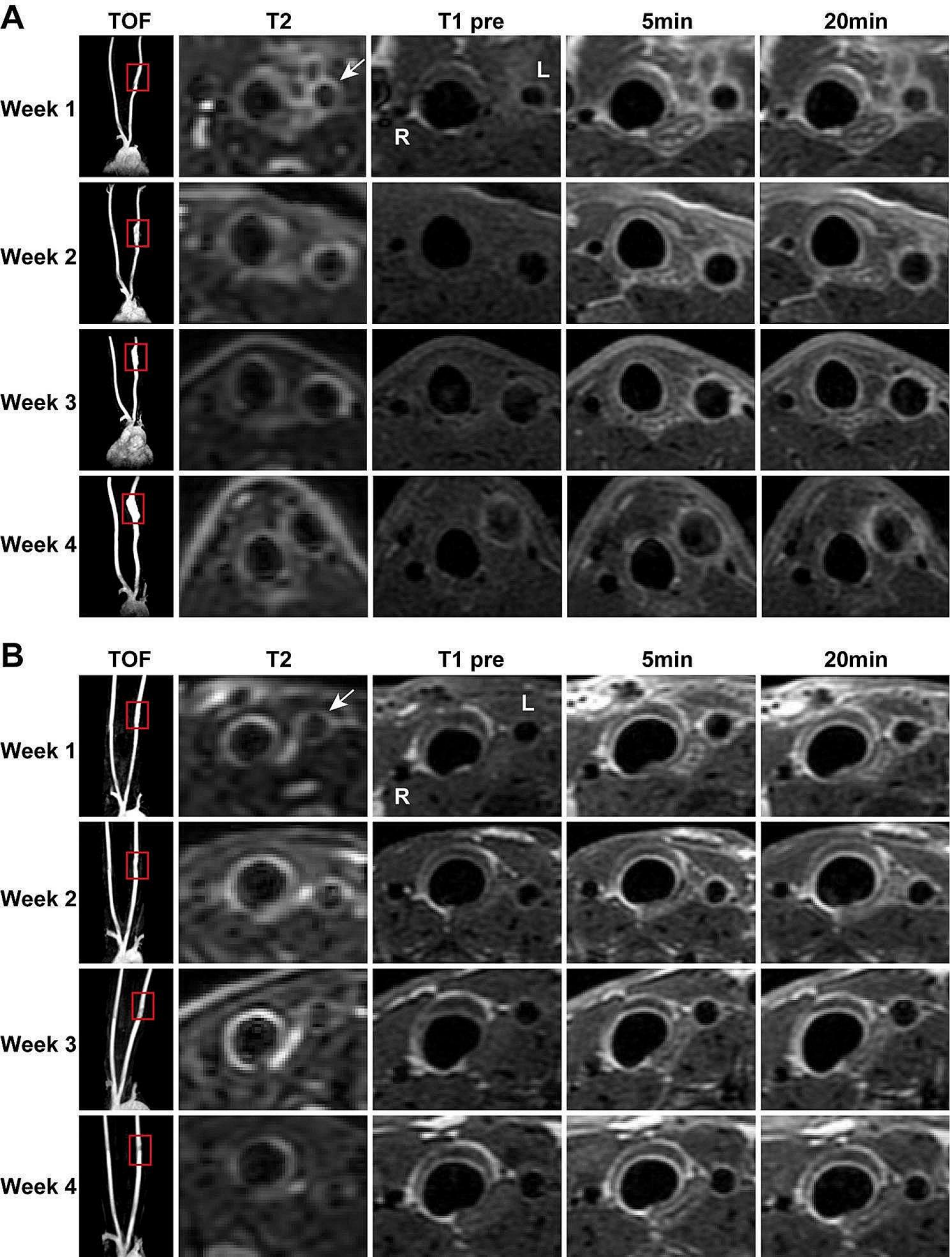
4-weeks Mn-TyrEDTA-enhanced imaging follow-up (3D TOF, precontrast T2-weighted and T1-weighted, and postcontrast T1-weighted MR images) on diseased arterial wall in elastase-induced rabbit carotid aneurysm. The diseased wall can be significantly enhanced by Mn-TyrEDTA in both ABAH(-) group ( *n* = 5) (**A**) and ABAH(+) group (*n* = 5) (**B**)

In this study, the aneurysm lesion was evaluated by 4-week follow-up MR imaging. The location and morphology of left carotid artery lesions can be well delineated on 3D TOF images (Fig. 3A and B). In the ABAH(-) group, the lesion size increased progressively from week 1 to 4 (Fig. 3, A), while the relevant change was hardly recognized in the ABAH(+) group during same period (Fig. 3, B).

On precontrast axial images, the diseased vascular wall in both groups showed high or low signal intensity on T2- or T1-weighted images, respectively, with limited resolution (Fig. 3, A and B). On postcontrast T1-weighted dynamic images, the diseased wall in both groups can be significantly enhanced by Mn-TyrEDTA, allowing visualization of aneurysm lesions and vessel diameter (Figs. 3, A and B and 5- and 20-min postcontrast).

**Fig. 4:**
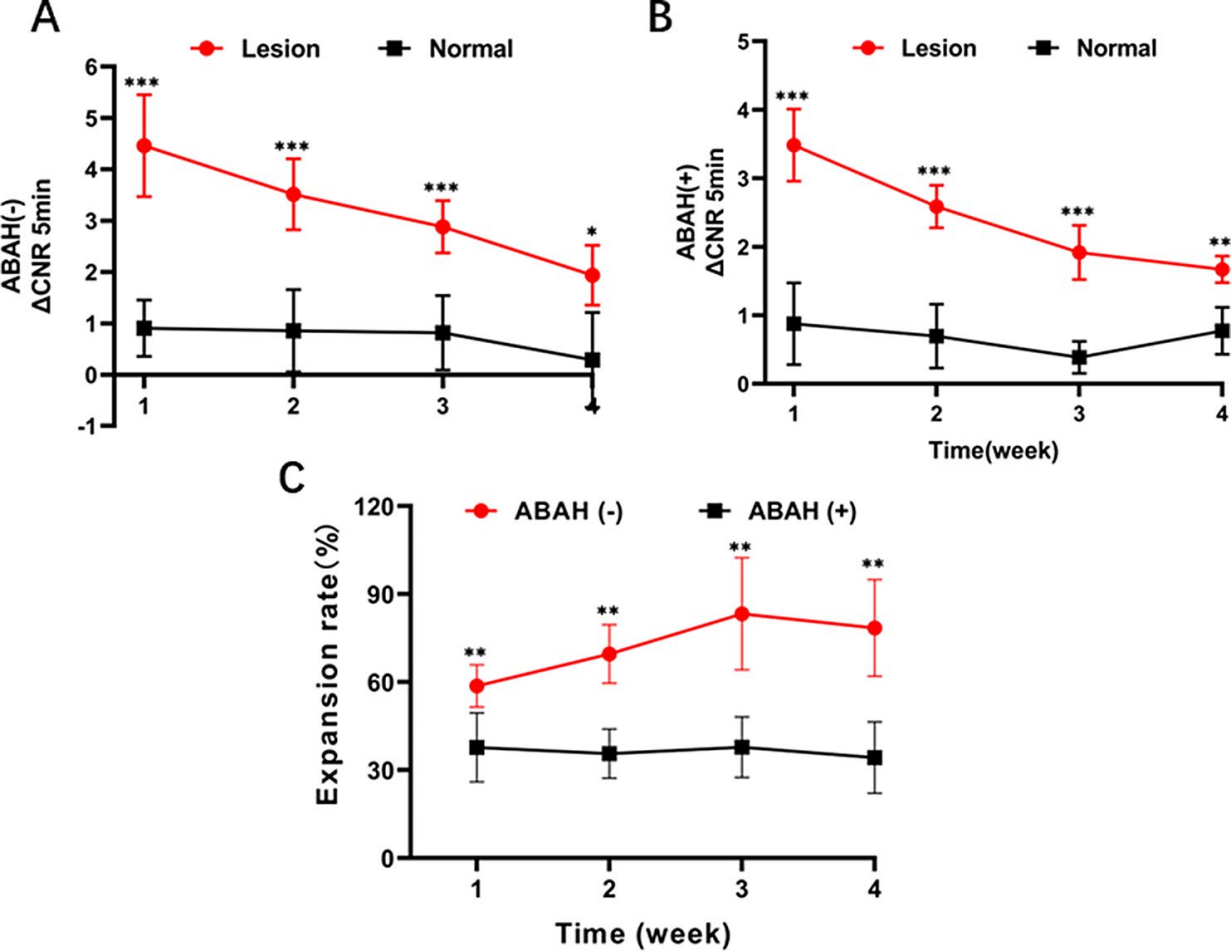
Analysis of the Mn-TyrEDTA-enhanced MR images of the diseased vascular wall of elastase-induced carotid aneurysm rabbit model in ABAH(-) and ABAH(+) groups. (**A**) The change in diseased artery (left)/ normal artery (right)-to-muscle contrast-to-noise ratio (∆CNR left/right-to-muscle) versus follow-up MR imaging time (up to 4 weeks) in ABAH(-) group; (**B**) The change in diseased artery (left)/ normal artery (right)-to-muscle contrast-to-noise ratio (∆CNR left/right-to-muscle) versus follow-up MR imaging time (up to 4 weeks) in ABAH(+) group; (**C**) The vascular expansion of aneurysm lesion versus ABAH-treated time (up to 4 weeks). A Student’s *t*-test was used for statistical analysis. The results are presented as the mean ± SD (*n* = 5. * *P* < 0.05, ** *P* < 0.01, *** *P* < 0.001 )

Quantitative analysis revealed that the change in lesion-to-control contrast ratio between diseased (left) and normal (right) arteries peaked at 5 min both in the ABAH(-) and ABAH(+) groups in the postcontrast dynamic imaging (Figs. S1 and S2). Therefore, we assessed the active state of inflammation over 4 weeks of ABAH treatment through the tracking of the change in ∆CNR _5 min-post_ (5 min post-injection of Mn-TyrEDTA). The Mn-TyrEDTA enhanced MRI showed weak ∆CNR (➔➔1) in normal vessel walls throughout the 4-week experiment both in ABAH(+) and ABAH(-) groups (Fig. 4, A and B). In contrast, the diseased aneurysm walls exhibited significantly higher ∆CNR compared to normal vessels in both groups. Notably, the ∆CNR in aneurysm walls was decreased in ABAH(+) group compared to the ABAH(-) group (4.56 ± 0.90 vs. 3.49 ± 0.88 at week 1; 3.51 ± 0.86 vs. 2.59 ± 0.70 at week 2; 2.88 ± 0.81 ± 1.92 ± 0.39 at week 3; 1.94 ± 0.29 vs. 1.67 ± 0.78 at week 4).

### Mn-TyrEDTA delineates different vascular expansion models of aneurysm lesion

Control arteries in both groups kept their normal morphology, but aneurysms in the ABAH(-) group showed significant growth over 4 weeks, with an expansion rate of 58.7 ± 7.25% in the first week, increasing to 69.62 ± 9.97% (week 2) and 83.26 ± 9.13% (week 3), then slightly decreasing to 78.45 ± 16.46% at the last week. In contrast, after intervention, aneurysms in the ABAH(+) group remained stable, with expansion rates significantly lower than the ABAH(-) group at all time points (37.74 ± 11.72% vs. 58.7 ± 7.25%, *p* < 0.01 at week 1; 35.62 ± 8.36% at week 2; 37.84 ± 10.35% at week 3; 34.26 ± 12.17% at week 4) (Fig. 4, C).

### ABAH attenuates vascular wall damages

**Fig. 5:**
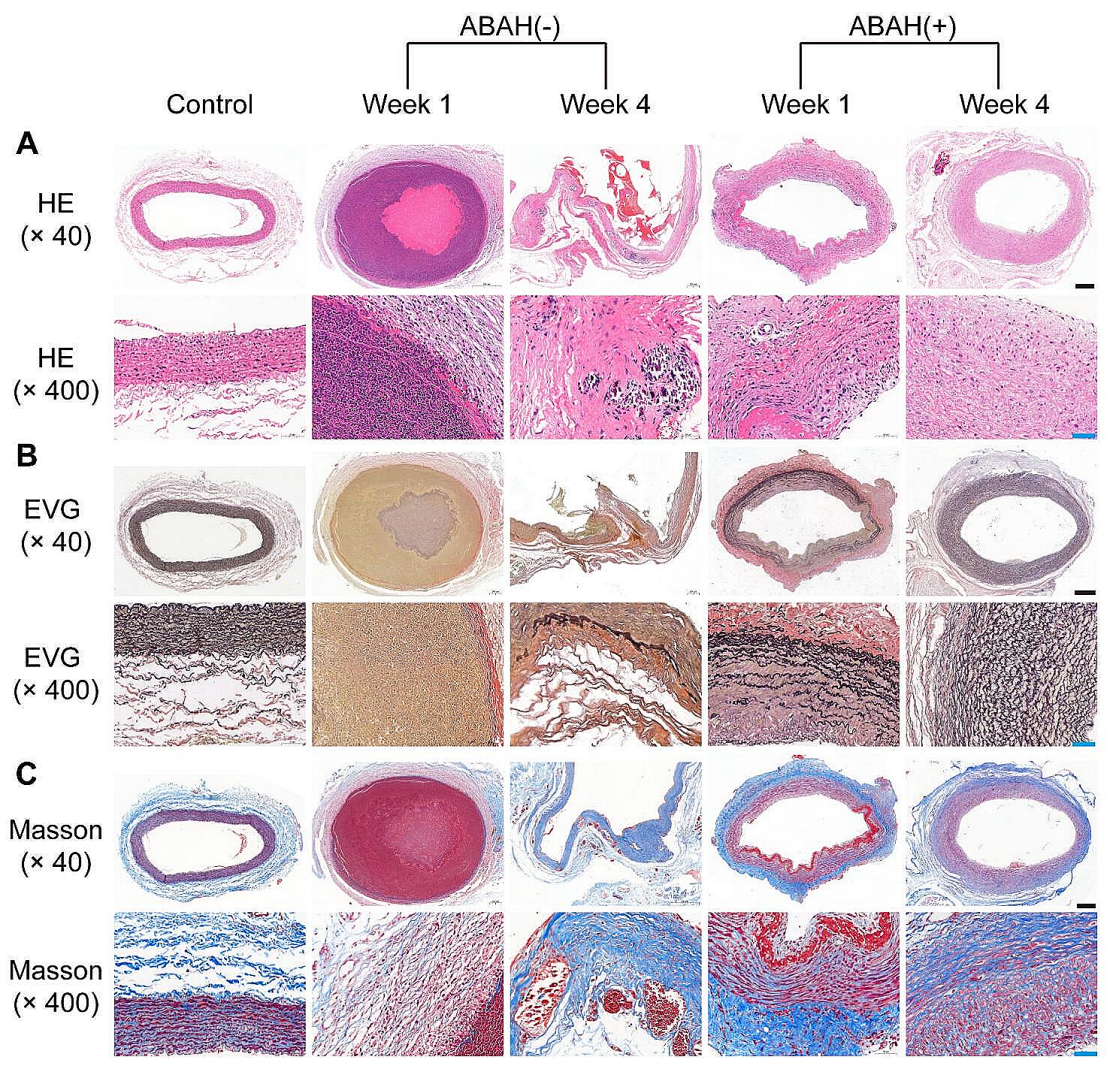
Histopathological changes of common carotid artery in rabbits. (**A**) HE staining. In ABAH(-) group showed the necrosis of vascular wall with loss of the internal elastic lamina, variable numbers of inflammatory cells infiltrated; (**B**) EVG staining. Elastic fibers in ABAH(-) group decreased obviously compared with control artery; (**C**) Masson staining. Collagen fiber was less than that of control artery in ABAH(-) group. Scale bar (black) = 200 μm. Scale bar (blue) = 50 μm

We evaluating inflammatory cells infiltration based on HE staining. Compared to the ABAH(-) group, ABAH treatment significantly inhibited inflammatory cells infiltration (including lymphocytes and neutrophils, 87 ± 7 vs. 1 ± 1 per high-power field, 15 ± 6 vs. 0 per high-power field, respectively) at week 1 (Table 1). Likewise, ABAH reduced elastin degradation ( Fig. 5, A and B) and fibrous tissue formation (Fig. 5, C).

**Table 1:** Neutrophils and macrophages were counted based on HE staining. Results are presented as the mean ± SD per high-power field, *n* = 3

Group	Inflammatory cells	
	Neutrophils	Macrophages
ABAH(+) Week 1	1 ± 1	0
ABAH(+) Week 4	1 ± 1	1 ± 1
ABAH(-) Week 1	87 ± 7	15 ± 6
ABAH(-) Week 4	7 ± 4	8 ± 6

At week 1, vessel wall integrity in the ABAH(+) group was largely unaltered compared to substantial loss of the internal elastic lamina (IEL) and tunica intima in the ABAH(-) group (Fig. 5, A). At week 4, pronounced vascular fibrosis occurred in both groups, but only the ABAH(-) group showed abundant calcification foci and poor elastin regeneration (Fig. 5, A)

### ABAH reduces the activity and expression of MPO in aneurysms

**Fig. 6:**
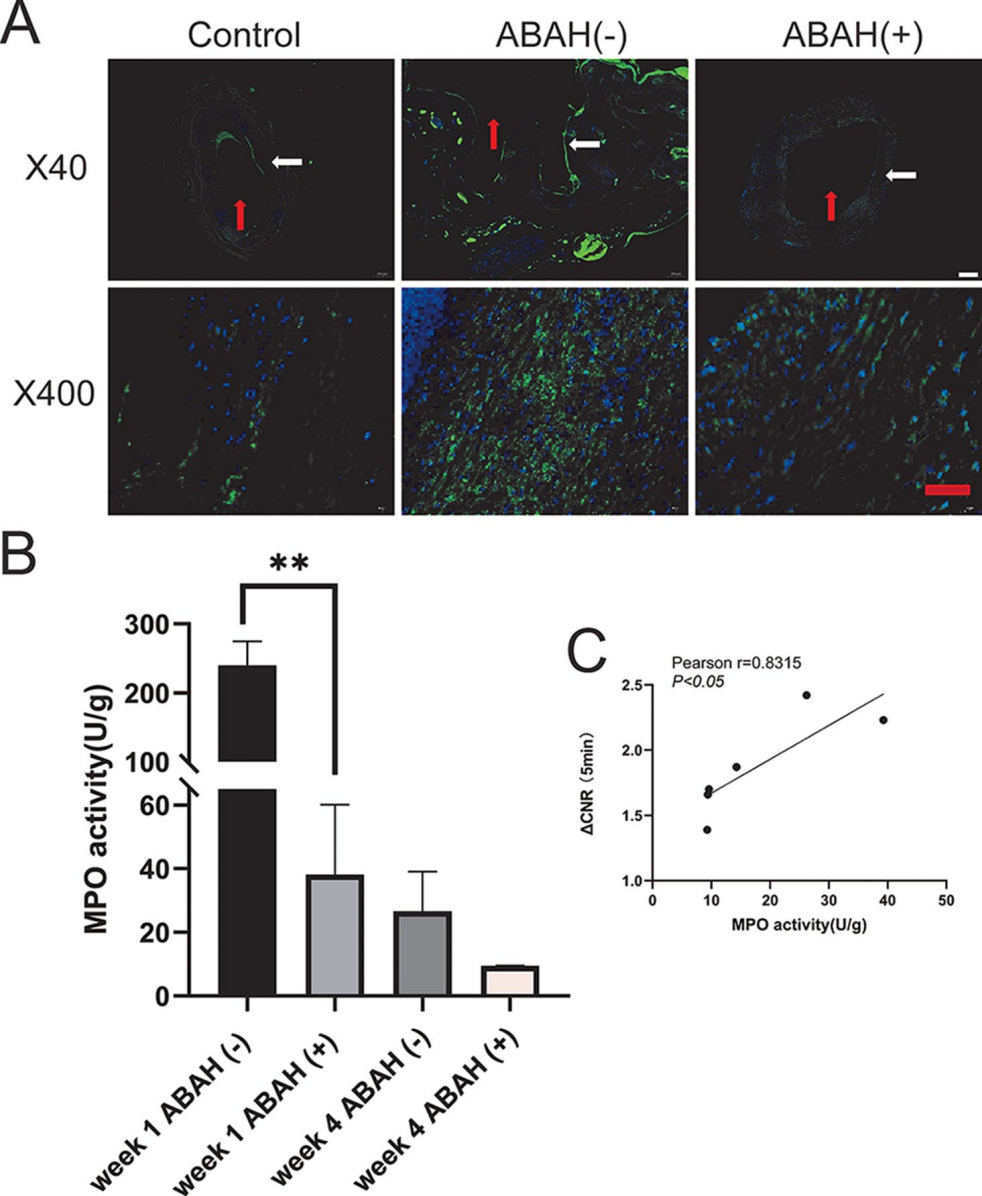
MPO immunofluorescence staining and MPO activity in rabbit carotid aneurysm tissues. (**A**) Immunofluorescence staining showed a lower level of positive expression of MPO in ABAH(+) group than that in ABAH(-) group both at week 1. Scale bar (white) = 200 μm. Scale bar (red) = 50 μm.). Arterial lumen (red arrow), arterial wall (white arrow); (**B**) Quantitative analysis of MPO activity revealed a significant lower level MPO activity of ABAH(+) group than that of the ABAH(-) group at week 1. A Student’s *t-*test was used for statistical analysis. The results are presented as the mean ± SD (*n* = 3. ** *P* < 0.01); (**C**) Relationship between the ΔCNR determined from the images of MRI and the MPO activity ex vivo at week 4. The linear regression lines are in black

Immunofluorescent staining showed significant low level of MPO expression in the control arteries, and lower level of MPO expression in the ABAH(+) group than the ABAH(-) group at week 1 was observed too (Fig. 6, A). Correspondingly, MPO activity assay revealed a significant 6.3-fold lower level in the ABAH(+) group (35.1 ± 22.0 vs. 240.0 ± 35.1 U/g protein, *n* = 3, *P* < 0.01) at week 1. MPO activity decreased in both groups at week 4, though no statistical significance (*P* > 0.05) (Fig. 6, B). To further investigate the correlation between MPO activity measured in vivo and ex vivo, we calculated the Pearson Correlation Coefficient between ΔCNR and MPO activity (guaiacol-based activity assay) at week 4, which showed a Pearson’s *r* = 0.8315, *P* < 0.05 (Fig. 6, C, strong correlation Pearson’s *r* > 0.5), indicating a strong correlation between contrast enhancement and ex vivo MPO activity.

### ABAH reduces the production of ROS in AHAH (+) group

**Fig. 7:**
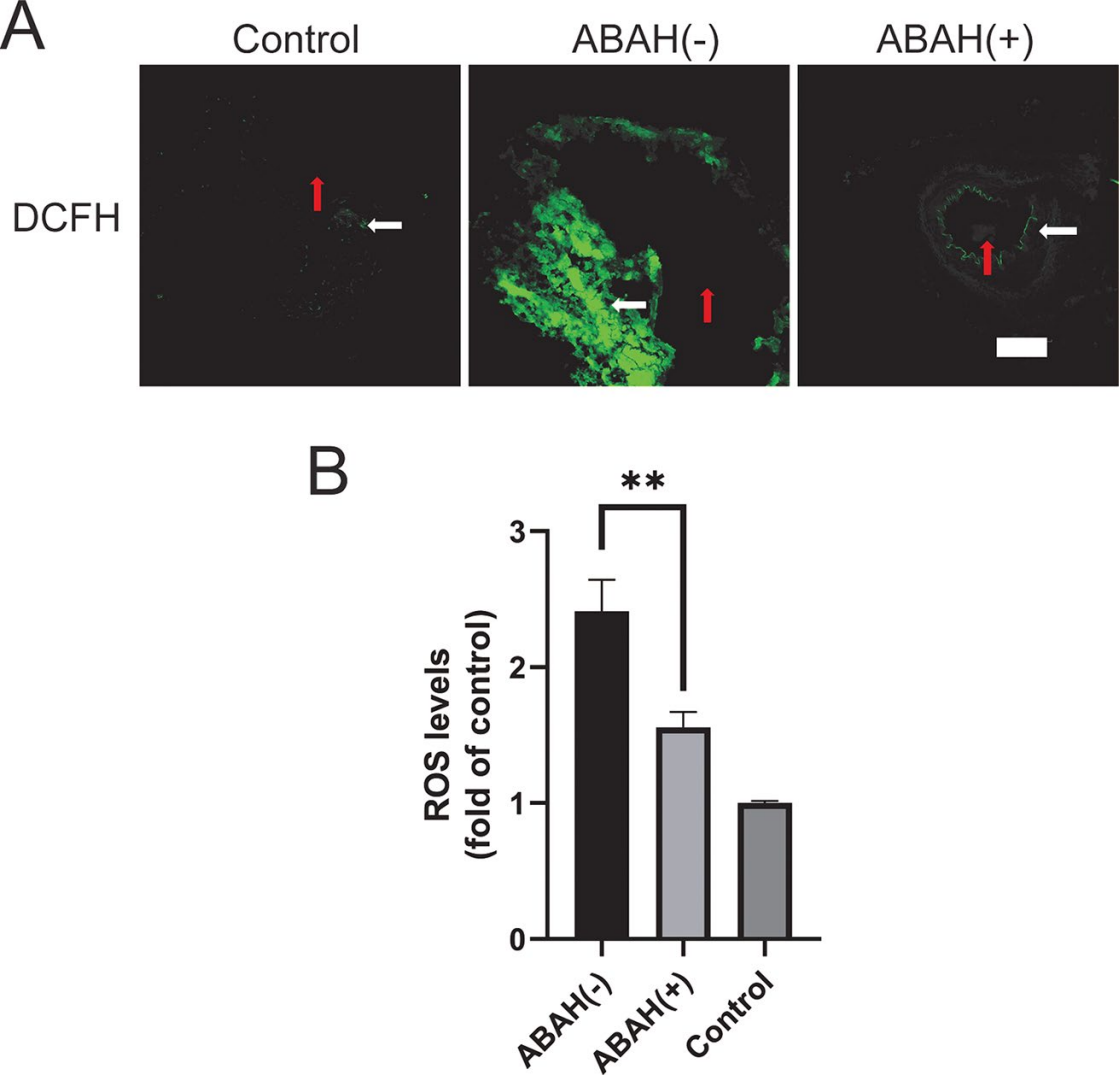
ABAH reduced the ROS production in ABAH (+) group. (**A**) level of ROS production was measured using a DCFH fluorescent probe. Scale bar = 200 μm. Arterial lumen (red arrow), arterial wall (white arrow); (**B**) Mean fluorescence intensity (MFI) of arterial wall was analyzed by ImageJ. The MFI of the control group was set to 1, and the MFI of the other groups was compared with that of the control group. Scale bar = 200 μm. A Student’s *t*-test was used for statistical analysis. The results are presented as the mean ± SD (*n* = 3. ** *P* < 0.01)

ROS levels in the aneurysm tissue sections were detected using DCFH via fluorescence microscopy (Fig. 7). As shown in Fig. 7A, the control artery showed negligible ROS level, and the ABAH(+) group displayed much lower ROS levels than the ABAH(-) group and the mean fluorescent intensity (MFI) fold change in the ABAH(-) compared to the ABAH(+) group was 1.5-fold higher (*p* < 0.01, *n* = 3, Fig. 7, B).

### ABAH decreases the activity of MMP-2 and MMP-9

**Fig. 8:**
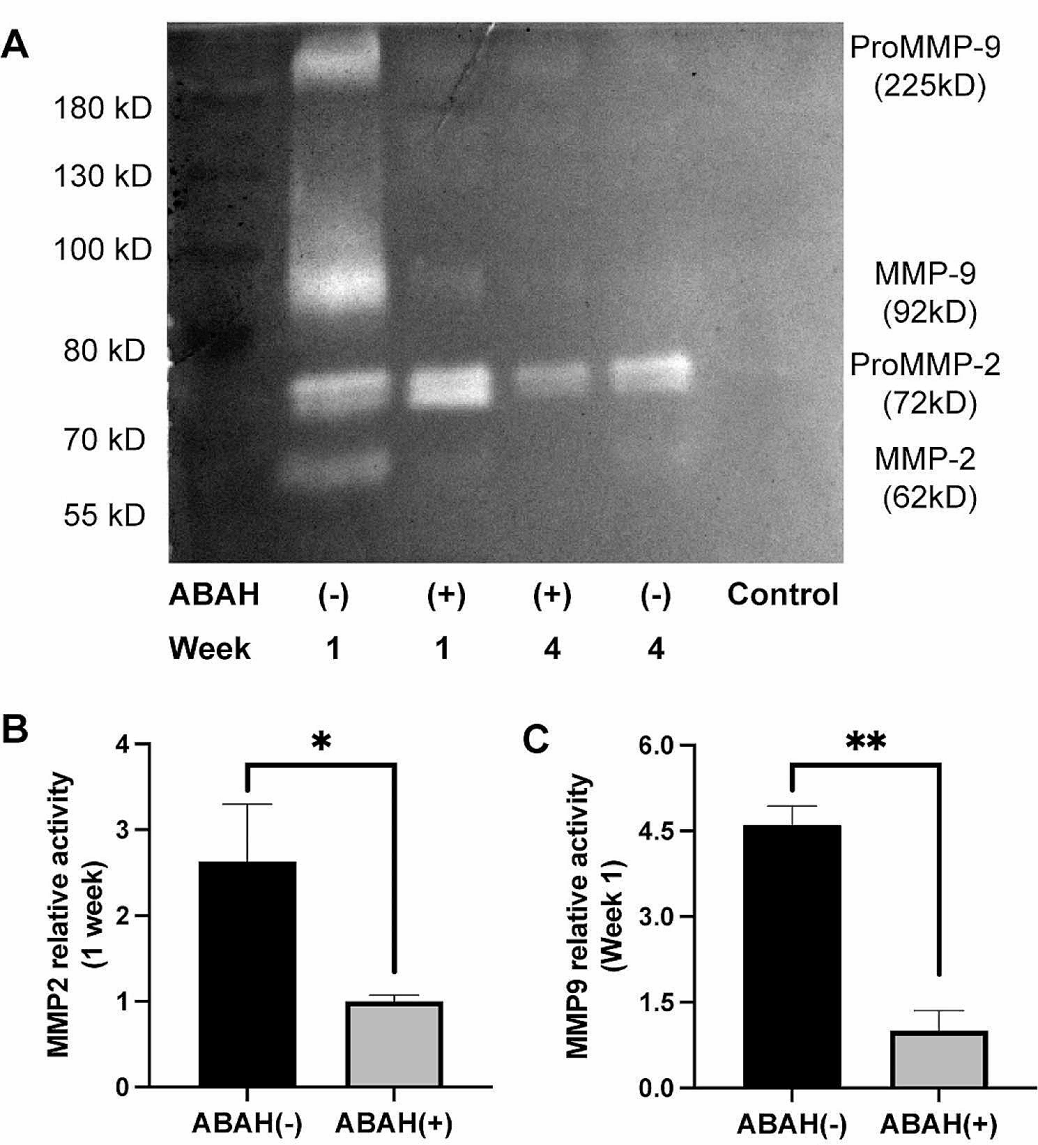
Activity of MMP-2 and MMP-9 in rabbit carotid aneurysms. (**A**)Typical zymogram analysis of MMP-9 and MMP-2 protein activities in ABAH(+) group and ABAH(-) group. (**B**) Quantitative analysis of relative activity of MMP-2 activity in each group. At week 1, MMP-2 activity was lower in ABAH(+) group; (**C**) Quantitative analysis of relative activity of MMP-9 activity in each group. At week 1, MMP-9 activity was significantly lower in ABAH(+) group. A Student’s *t*-test was used for statistical analysis. The results are presented as the mean ± SD (*n* = 3. * *P* < 0.05, ** *P* < 0.01)

Compared to the ABAH(-) group, MMP-2 and MMP-9 activity was significantly lower in the ABAH(+) group at week 1 (MMP-2: 2.6-fold,*P* < 0.05; MMP-9: 4.6-fold, *P* < 0.01) (Fig. 8). No significant difference in MMP-2 and MMP-9 activity was observed between two groups at week 4 (data not shown).

## Discussion

While the etiology of IA remains debate, inflammation-driven oxidative tissue damages decisively contributes to IA pathogenesis, as evidenced by infiltration of neutrophils, macrophages and other inflammatory cells at lesion site [8,9,22]. To explore noninvasive, dynamic IA monitoring based on inflammation’s key role, we focused on MPO, a hallmark of neutrophils and macrophages activation and function [38,39], to investigate if it could serve as an imaging biomarker and therapeutic target.

Most recently, King et al. evaluated a ROS activated probe Fe-PyC3A as a tool for MRI of inflammation in a rabbit model of saccular aneurysm, and found that enhancement ratios values measured immediately after dynamic MRI (20 min post-injection) were significantly higher in the case of Fe-PyC3A (1.25 ± 0.06) than for gadobutrol injection (1.03 ± 0.03) [40]. However, Fe-PyC3A did not directly reflect the activity of MPO. In this work, we monitored dynamic aneurysm changes over 4 weeks using MPO-sensitive MRI with the Mn-TyrEDTA probe to track the active state of inflammation. We found that carotid arterial wall in the ABAH(+) group were less enhanced by Mn-TyrEDTA compared with that in the ABAH(-) group, indicating reduced activity of MPO. This was supported by histology (less neutrophils and macrophages in the ABAH(+) group at week 1), MPO activity assays (6.3-fold lower in the ABAH(+) group at week 1), and MPO immunofluorescence (lower MPO expression in the ABAH(+) group). These results show Mn-TyrEDTA can effectively and directly detect MPO activity in aneurysmal lesion, indicating potential for noninvasive, dynamic IA inflammation monitoring.

Mn-TyrEDTA enhanced MRI revealed significantly higher MPO activity in aneurysm walls compared to normal vessel walls, but unfortunately, the difference in ∆CNR between the ABAH(+) and ABAH(-) groups was not as significant as expected. This indicates that the detected MPO activity using Mn-TyrEDTA after ABAH inhibition may be suboptimal. A potential reason is the rapid blood clearance of Mn-TyrEDTA, as reported previously [26], leading to insufficient probe retention and detection sensitivity. To address this limitation, activatable MPO-targeting probes could be explored, such as the MPO-sensitive probes developed by Wang et al. [41–43] which undergoes hydrophobic modification to enable accumulation at sites of MPO activation.

More interestingly, inhibiting MPO by ABAH prevented the progression of aneurysms in our study, as evidenced by MRA-based expansion rate measurement, MMPs activity assays and Histology study. Microscopically, MPO-mediated oxidative damage appeared ameliorated in the ABAH(+) group at week 1, with less arterial wall necrosis and internal elastic lamina degradation. While both groups showed fibrous repair of vascular remodeling, massive calcification only in the ABAH(-) group at week 4 suggests earlier severe damage. Importantly, the ABAH(+) group expansion rate remained < 50% throughout the study, not even meeting aneurysm diagnostic criteria [44].

Possibly due to effective MPO inhibition by ABAH, ROS production, MMP-2 and MMP-9 activity was significantly lower in ABAH (+) group, resulting in less internal elastic lamina and collagen loss at week 1. Overall, we showed MPO inhibition can reduce arterial wall damage and prevent aneurysms progression in a preclinical model, consistent with other studies. For example, Kim et al. found MPO gene deletion or taurine (which scavenges MPO-generated oxidants) prevents abdominal aortic aneurysm formation [45].

We established a model of the relatively rare fusiform IA [46,47] which shows circumferential carotid dilation and is surgically challenging to treat due to lack of a defined neck [48]. Fusiform IAs share some histological features with saccular IAs, including internal elastic lamina loss, tunica media degradation, and tunica intima hyperplasia [49]. Most importantly, we also found neutrophils and macrophages enrichment at lesion sites, as in saccular IAs. Regardless of IA type (fusiform vs. saccular) or location (intracranial, thoracic, abdominal), inflammation-driven tissue damage is central to IA formation, recruiting inflammatory cells like neutrophils, macrophages, T cells, and B cells to the tunica media and adventitia [50–53]. Therefore, Mn-TyrEDTA could assess the inflammatory response in various IAs, though further study is needed.

## Limitation

Certain limitations of this study should be acknowledged. First, the sample size was relatively small. Second, MMPs expression could not be assessed due to lack of rabbit-compatible anti-MMP-2 or MMP-9 antibodies. Third, the direct link between MPO and MMPs/TIMPs activity was not demonstrated in the results. Fourth, tests or control experiments to validate methodology of IF were not conducted. Finally, the model used to simulate complex human aneurysm pathology has inherent limitations. However, given inflammation’s significant role in aneurysms, these limitations are unlikely to impact the overall conclusions.

## Conclusion

This study demonstrated that MPO plays an important role in the formation and progression of elastase-induced carotid aneurysms. Inhibition of MPO by ABAH attenuates multiple pathological changes in the aneurysmal walls, including inflammation, elastin degradation, ROS production and matrix metalloproteinase activation. Mn-TyrEDTA enhanced MRI showed promise as a noninvasive tool for monitoring aneurysm inflammation and the effects of MPO inhibition.

### Electronic supplementary material

Below is the link to the electronic supplementary material.

Supplementary Material 1

## Data Availability

The datasets used or analysed during the current study are available from the corresponding author on reasonable request.

## Abbreviations

3D TOF: three-dimensional time of flight
ABAH: 4-aminobenzoic acid hydrazide
CNR: contrast-to-noise ratio
ECM: extracellular matrix
EVG: Elastin Van Gieson
FA: flip angle
FOV: field of view
HE: hematoxylin and eosin
HOCl: hypochlorous acid
IA: intracranial aneurysm
MMPs: matrix metalloproteinases
MPO: myeloperoxidase
NEX: number of excitations
ROI: regions of interest
ROS: reactive oxidative species
SD: standard deviation
SI: signal intensity
T2W: T2-weighted
TIMPs: tissue inhibitors of matrix metalloproteinases
VSMCs: vascular smooth muscle cell
ΔCNR: changes in contrast-to-noise ratio
ΔOD: optical density change
