# Supplementary material for: Targeting myeloperoxidase to stabilize unruptured aneurysm: an imaging-guided approach
Source: BMC Cardiovasc Disord. 2024 Mar 20;24:169. doi: 10.1186/s12872-024-03822-1 (PMC10953282; doi:10.1186/s12872-024-03822-1)
Supplement: Supplementary file 1 — Supplementary Material 1 [file 12872_2024_3822_MOESM1_ESM.docx]

# Targeting Myeloperoxidase to Stabilize Unruptured Aneurysm: An Imaging-Guided Approach

Xingchi, Shi^1,2^; Huiyu, Wu^1,3^;Yuan, Xue^1,4^; ;Chenyi, Shen^4^; Lei, Zhong^1^; Jun, Lei ^3^; Zhiyang, Xia^4*^; Ying, Yang^1,2*^; Jiang, Zhu^1,3*^

From the ^1^Medical Imaging Key Laboratory of Sichuan province, Department of Oncology, Affiliated Hospital of North Sichuan Medical College, Maoyuan Road 1, Nanchong City, Sichuan, 637000, China.

^2^Department of Cardiovascular disease, School of Clinical Medicine, Affiliated Hospital of North Sichuan Medical College, Maoyuan Road 1, Nanchong City, Sichuan, 637000, China.

^3^School of Pharmacy, North Sichuan Medical College, Fujiang Road 234, Nanchong City, Sichuan, 637000, China

^4^Institute of Basic Medicine and Forensic Medicine, North Sichuan Medical College, Fujiang Road 234, Nanchong City, Sichuan, 637000, China

* Corresponding Author(s): Zhiyang Xia, [Xiazhiyang@nsmc.edu.cn](mailto:Xiazhiyang@nsmc.edu.cn); Ying Yang, [yangying@nsmc.edu.cn](mailto:yangying@nsmc.edu.cn); Jiang Zhu, [zhujiang@nsmc.edu.cn](mailto:zhujiang@nsmc.edu.cn)


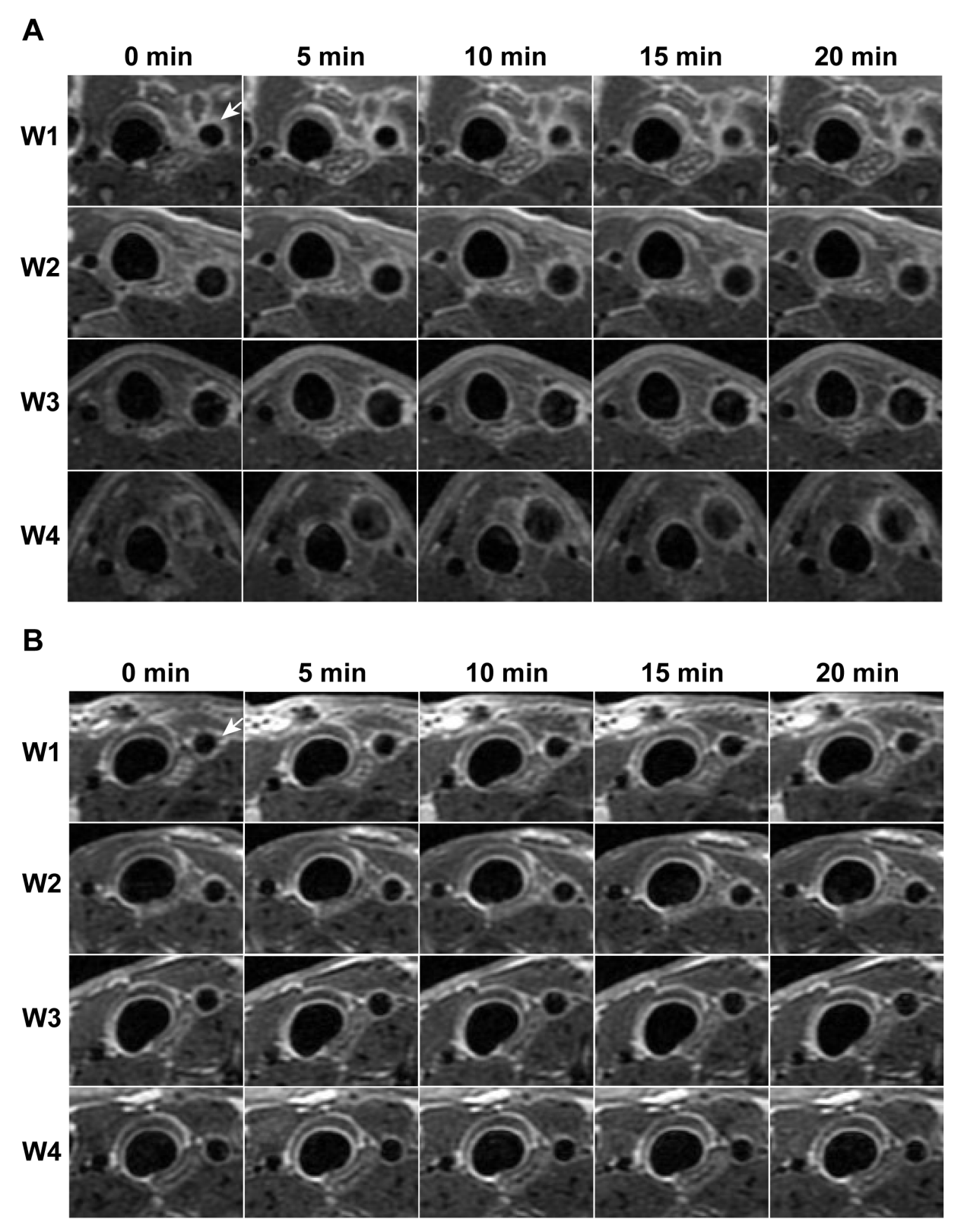


Figure S1 4-weeks MPO-enhanced imaging follow-up (postcontrast T1-weighted MR images) on diseased arterial wall (white arrow) in elastase-induced rabbit carotid aneurysm. The diseased wall can be significantly enhanced by MPO-Mn in both ABAH(-) group ( n = 5) (A) and ABAH(+)group(n = 5) (B).


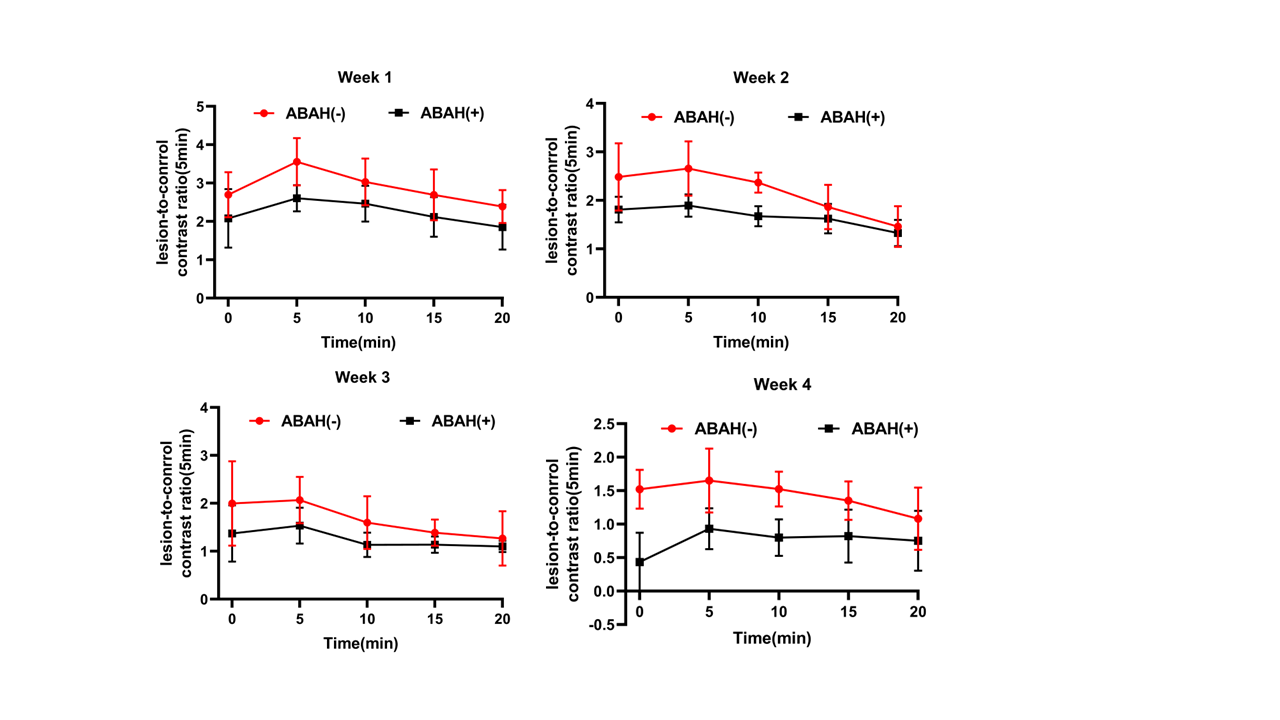


Figure S2 The change in lesion-to-control contrast ratio versus ABAH-treated time in dynamic MPO-Mn enhanced MR imaging, lesion-to-control contrast ratio peaked at 5 minutes both in ABAH(-) and ABAH(+) groups throughout this study.

Table S1 Raw data of ∆CNR 5min for individual rabbit.

| ABAH(-)∆CNR 5min | | Rabbit 1 | Rabbit 2 | Rabbit 3 | Rabbit 4 | Rabbit 5 |
| --- | --- | --- | --- | --- | --- | --- |
| Week 1 | ΔCNR_lesion | 4.5025 | 3.835 | 6.0925 | 3.6925 | 5.1175 |
|  | ΔCNR_Normal | 0.94 | 0.5075 | 1.32 | 0.435 | 1.77 |
| Week 2 | ΔCNR_lesion | 3.945 | 4.1125 | 3.7775 | 3.1575 | 3.8125 |
|  | ΔCNR_Normal | 1.9975 | 1.3175 | 0.17 | 0.4025 | 1.2925 |
| Week 3 | ΔCNR_lesion | 2.7625 | 2.475 | 3.61 | 3.0875 | 2.2 |
|  | ΔCNR_Normal | 0.7625 | 0.665 | 1.4925 | 0.3125 | -0.155 |
| Week 4 | ΔCNR_lesion | 1.5175 | 2.23 | 2.615 | 1.385 | 2.17 |
|  | ΔCNR_Normal | -0.4125 | 1.2825 | 0.8625 | -0.5825 | 0.3534 |
| ABAH(+)∆CNR 5min | | Rabbit 1 | Rabbit 2 | Rabbit 3 | Rabbit 4 | Rabbit 5 |
| Week 1 | ΔCNR_lesion | 3.13 | 2.8775 | 3.3625 | 3.955 | 4.1025 |
|  | ΔCNR_Normal | 0.315 | 0.525 | 0.6175 | 1.8025 | 1.1275 |
| Week 2 | ΔCNR_lesion | 2.3625 | 2.915 | 2.2525 | 2.4925 | 2.9225 |
|  | ΔCNR_Normal | 0.3175 | 1.42 | 0.375 | 0.4575 | 0.9175 |
| Week 3 | ΔCNR_lesion | 1.6975 | 2.0675 | 1.72 | 2.545 | 1.5625 |
|  | ΔCNR_Normal | 0.1125 | 0.3325 | 0.2275 | 0.6225 | 0.635 |
| Week 4 | ΔCNR_lesion | 1.7025 | 1.39 | 1.6675 | 1.9425 | 1.66 |
|  | ΔCNR_Normal | 1.245 | 0.345 | 0.5575 | 0.8675 | 0.86 |
